# Clustering of cardiovascular risk factors and carotid intima-media thickness: The USE-IMT study

**DOI:** 10.1371/journal.pone.0173393

**Published:** 2017-03-21

**Authors:** Xin Wang, Geertje W. Dalmeijer, Hester M. den Ruijter, Todd J. Anderson, Annie R. Britton, Jacqueline Dekker, Gunnar Engström, Greg W. Evans, Jacqueline de Graaf, Diederick E. Grobbee, Bo Hedblad, Suzanne Holewijn, Ai Ikeda, Jussi Kauhanen, Kazuo Kitagawa, Akihiko Kitamura, Sudhir Kurl, Eva M. Lonn, Matthias W. Lorenz, Ellisiv B. Mathiesen, Giel Nijpels, Shuhei Okazaki, Joseph F. Polak, Jacqueline F. Price, Christopher M. Rembold, Maria Rosvall, Tatjana Rundek, Jukka T. Salonen, Matthias Sitzer, Coen D. A. Stehouwer, Tomi-Pekka Tuomainen, Sanne A. E. Peters, Michiel L. Bots

**Affiliations:** 1 Julius Center for Health Sciences and Primary Care, University Medical Center Utrecht,Utrecht, the Netherlands; 2 Laboratory of Experimental Cardiology, University Medical Center Utrecht, Utrecht, the Netherlands; 3 Department of Cardiac Sciences and Libin Cardiovascular Institute of Alberta, University of Calgary, Alberta, Canada; 4 Department of Epidemiology and Public Health University College London, London, United Kingdom; 5 Institute for Health and Care Research, VU University Medical Center, Amsterdam, The Netherlands; 6 Institute of Medicine, University of Gothenburg, Gothenburg, Sweden; 7 Department of Biostatistical Sciences and Neurology, Wake Forest School of Medicine, Winston-Salem, NC, United States of America; 8 Department of General Internal Medicine, Division of Vascular Medicine, Radboud University Medical Center, Nijmegen, the Netherlands; 9 University of Malaya Medical Center, Kuala Lumpur, Malaysia; 10 Department of General Internal Medicine, Division of Vascular Medicine, Radboud University Medical Center, Nijmegen, the Netherlands; 11 Osaka Medical Center for Health Science and Promotion, Osaka, Japan; 12 The Institute of Public Health and Clinical Nutrition, University of Eastern Finland, Kuopio, Finland; 13 Department of Neurology, Tokyo Women Medical University, Tokyo, Japan; 14 Department of Medicine, Division of Cardiology and Population Health Research Institute, McMaster University, Hamilton, Ontario, Canada; 15 Department of Neurology, University Hospital, Goethe-University, Frankfurt am Main, Germany; 16 Department of Neurology, Krankenhaus Nordwest, Frankfurt am Main, Germany; 17 Brain and Circulation Research Group, Department of Clinical Medicine, University of Tromsö, Tromsö, Norway; 18 Department of Neurology, National Cerebral and Cardiovascular Center, Osaka, Japan; 19 Department of Radiology, Tufts University School of Medicine, Boston, United States of America; 20 Usher Institute, University of Edinburgh, Edinburgh, United Kingdom; 21 Cardiology Division, Department of Internal Medicine, University of Virginia, Charlottesville, VA, United States of America; 22 Department of Neurology, Miller School of Medicine, University of Miami, Miami, Fl, United States of America; 23 Department of Public Health, Faculty of Medicine, University of Helsinki, Helsinki, Finland; 24 MAS-Metabolic Analytical Services Oy, Helsinki, Finland; 25 Department of Neurology, University Hospital, Goethe-University, Frankfurt am Main, Germany and Department of Neurology Klinikum Herford, Germany; 26 Department of Internal Medicine and Cardiovascular Research Institute Maastricht, Maastricht University Medical Center, Maastricht, The Netherlands; 27 The George Institute for Global Health, University of Oxford, Oxford, United Kingdom; Medizinische Universitat Innsbruck, AUSTRIA

## Abstract

**Background:**

The relation of a single risk factor with atherosclerosis is established. Clinically we know of risk factor clustering within individuals. Yet, studies into the magnitude of the relation of risk factor clusters with atherosclerosis are limited. Here, we assessed that relation.

**Methods:**

Individual participant data from 14 cohorts, involving 59,025 individuals were used in this cross-sectional analysis. We made 15 clusters of four risk factors (current smoking, overweight, elevated blood pressure, elevated total cholesterol). Multilevel age and sex adjusted linear regression models were applied to estimate mean differences in common carotid intima-media thickness (CIMT) between clusters using those without any of the four risk factors as reference group.

**Results:**

Compared to the reference, those with 1, 2, 3 or 4 risk factors had a significantly higher common CIMT: mean difference of 0.026 mm, 0.052 mm, 0.074 mm and 0.114 mm, respectively. These findings were the same in men and in women, and across ethnic groups. Within each risk factor cluster (1, 2, 3 risk factors), groups with elevated blood pressure had the largest CIMT and those with elevated cholesterol the lowest CIMT, a pattern similar for men and women.

**Conclusion:**

Clusters of risk factors relate to increased common CIMT in a graded manner, similar in men, women and across race-ethnic groups. Some clusters seemed more atherogenic than others. Our findings support the notion that cardiovascular prevention should focus on sets of risk factors rather than individual levels alone, but may prioritize within clusters.

## Introduction

Coronary heart disease and stroke are among the largest contributors of years of life lost and disability adjusted life years in both developed and developing countries [1]. The burden of cardiovascular events is to a large extent preventable through modification of cardiovascular risk factors [2,3]. Risk factors such as elevated blood pressure, smoking, overweight and elevated total cholesterol have been identified as being among the top ten factors responsible for loss of disability adjusted life years [1]. Atherosclerosis underlies the occurrence of a major part of the cardiovascular burden[4]. The development of atherosclerosis starts at a young age, and slowly progresses with ageing [5]. Prevention of the development and progression of atherosclerosis therefore may prevent cardiovascular events from occurring. There is a wealth of evidence supporting the relation of a single risk factor level with presence and extent of atherosclerosis. Although we know that risk factors tend to cluster within individuals [6], studies addressing the relation between clusters of risk factors and atherosclerosis are limited [7], most dealing with the metabolic syndrome as a cluster. Yet, some risk factor clusters may be more atherogenic than others, and the importance of the clusters may vary across groups of individuals, which then may lead to different approaches to prevent cardiovascular disease, in particular when resources are limited. We assessed the relation between clusters of two, three, or four risk factors and atherosclerosis, as measured by common carotid intima-media thickness, in the general population, and compared these relations between men and women, and across race-ethnic groups.

## Methods

### Study population

The present cross-sectional analyses are based on baseline data from the cohort participating in the USE-IMT collaboration, an individual participant data meta-analysis established to determine the incremental value of measuring common carotid intima media thickness (CIMT) in predicting cardiovascular events [8]. Population-based prospective cohort studies with data on cardiovascular risk factors, common CIMT, and follow-up for cardiovascular events were identified through systematic literature search and expert recommendation. In the current analysis, we included 59,025 individuals from 14 studies (Table 1) [9–22]. Race-ethnic groups were categorised as White, Black, Hispanic or Asian.[23] Diabetes mellitus was defined using the definitions of the individual cohorts, that is using questionnaire information, and /or use of blood glucose lowering medication or fasting or casual glucose level.[24] For the definition of history of cardiovascular disease, study-specific definitions were used[8].

**Table 1:** Baseline characteristics of USE-IMT cohorts in the present analysis.

Study	Individuals	Age (years)	Gender(% male)	Mean CIMT (mm)	BMI (kg/m2)	Smoking (% yes)	SBP (mmHg)	DBP (mmHg)	TC (mmol/L)	HDL (mmol/L)	LDL (mmol/L)	Glucose (mmol/L)
Malmo	5163	57.5 (5.9)	40.5	0.77 (0.15)	25.6 (3.8)	22.5	141 (19)	87 (9)	6.2 (1.1)	1.4 (0.4)	4.2 (1.0)	5.2 (1.4)
CAPS	5056	50.1 (13.1)	48.9	0.73 (0.16)	26.6 (4.1)	20.9	128 (17)	77 (10)	5.7 (1.1)	1.5 (0.4)	3.4 (0.9)	NA
KIHD	1399	52.4 (6.4)	100.0	0.78 (0.18)	26.6 (3.5)	40.1	132 (17)	88 (10)	5.8 (1.0)	1.3 (0.3)	3.9 (1.0)	4.7 (1.3)
ARIC	15732	54.2 (5.7)	44.8	0.66 (0.15)	27.7 (5.4)	26.2	121 (19)	74 (11)	5.6 (1.1)	1.3 (0.4)	3.6 (1.0)	6.0 (2.3)
Virginia	741	56.9 (12.3)	54.8	0.82 (0.18)	26.4 (4.6)	7.3	139 (19)	84 (11)	5.8 (1.3)	1.2 (0.4)	3.7 (1.1)	NA
Tromso	6687	60.2 (10.2)	49.4	0.79 (0.16)	26.0 (4)	31.8	145 (23)	83 (13)	6.8 (1.3)	1.5 (0.4)	4.9 (1.2)	4.9 (1.3)
FATE	1578	49.4 (9.9)	99.7	0.72 (0.18)	28.5 (3.6)	12.0	128 (17)	82 (10)	5.3 (1.0)	1.2 (0.3)	3.3 (0.9)	5.3 (1.0)
OSACA2	769	65.8 (9)	59.2	0.91 (0.31)	23.1 (3.1)	23.3	137 (19)	79 (12)	5.4 (0.9)	1.5 (0.4)	3.0 (0.7)	5.9 (1.7)
MESA	6814	62.2 (10.2)	47.2	0.76 (0.18)	28.3 (5.5)	13.1	127 (21)	72 (10)	5.0 (0.9)	1.3 (0.4)	3.1 (0.8)	5.4 (1.7)
Hoorn	647	70.0 (6.5)	48.8	0.87 (0.17)	27.3 (4)	15.5	142 (21)	83 (11)	5.7 (1.0)	1.4 (0.4)	3.6 (0.9)	6.2 (1.4)
EAS	1115	69.0 (5.6)	49.8	0.77 (0.28)	25.6 (3.8)	18.8	147 (24)	82 (12)	7.1 (1.3)	1.5 (0.4)	5.3 (1.2)	5.8 (1.3)
NOMAS	1770	69.4 (9.3)	39.9	0.73 (0.09)	28.1 (5)	16.1	141 (20)	83 (11)	5.2 (1.0)	1.2 (0.4)	3.3 (0.9)	5.7 (2.4)
NBS	1246	60.7 (5.8)	46.5	0.83 (0.11)	26.5 (3.9)	16.1	128 (15)	78 (10)	5.9 (1.0)	1.4 (0.4)	3.8 (0.9)	5.2 (0.9)
Whitehall	10308	61.1 (6.0)	66.9	0.79 (0.16)	26.8 (4.4)	8.5	128 (17)	74 (11)	5.7 (1.0)	1.6 (0.5)	3.5 (1.0)	NA
Combined	59025	58.0 (9.6)	52.5	0.74(0.17)	27.0 (4.7)	20.5	130 (21)	77 (11)	5.8 (1.2)	1.4 (0.4)	3.7 (1.1)	5.5 (1.8)

ARIC: Atherosclerosis Risk in Communities Study; CAPS: Carotid Atherosclerosis Progression Study; EAS: Edinburgh Artery Study; FATE: The Firefighters and Their Endothelium Study; Hoorn: The Hoorn Study; KIHD: Kuopio Ischaemic Heart Disease Risk Factor Study; MESA; Multi-race/ethnic Study of Atherosclerosis; NBS: Nijmegen Biomedical Study 2; NOMAS: Northern Manhattan Study; OSACA2: Osaka Follow-Up Study for Carotid Atherosclerosis 2; Tromso: Tromso Study; Whitehall: Whitehall II Study; CIMT: mean common carotid intima media thickness; BMI, body mass index; SBP, systolic blood pressure; DBP, diastolic; TC, total cholesterol; HDL, high-density lipoprotein; LDL, low-density lipoprotein.

### Cardiovascular risk factor definition

Methods of measurement of baseline risk factors have been described in previous studies[9–22]. Smoking status was ascertained from self-report questionnaires and defined as current smoking. For each individual, body mass index (BMI) was calculated from measured body weight (in kilograms) divided by measured height (in meters) squared. Overweight was defined as having a BMI ≥ 25 kg/m^2^ [25]. Elevated blood pressure was defined as systolic blood pressure (SBP) ≥ 140 mmHg or diastolic blood pressure (DBP) ≥ 90mmHg[26], and elevated cholesterol was defined as total cholesterol ≥ 6.2 mmol/L[27], irrespective of the use of medication. Clusters with diabetes were not included due to the low prevalence (8.5%) in this study population. Based on these risk factors, we defined 15 separate groups ranging from no risk factor present, one risk factor present (only high BP, only smoking, only high cholesterol, only overweight), two risk factors (BP-smoking; BP-overweight; overweight-smoking; cholesterol-overweight; cholesterol-smoking; cholesterol-blood pressure), three risk factors (cholesterol-BP-smoking; overweight-BP-smoking; cholesterol-overweight-BP; cholesterol-overweight-smoking) and four risk factors present (cholesterol-overweight-BP-smoking).

### Common carotid intima-media thickness

For each cohort, average mean common CIMT was calculated for each individual using the maximum information of measurements from carotid angles, near and/or far wall measurements, and left and or right side measurements.[8]. Incomplete data on common CIMT, cardiovascular risk factors, and (time to) events resulted in 12% missing data points, which were imputed using single imputation for each cohort separately (using the Multivariate Imputation by Chained Equations package of R). Predictors in our imputation model included all variables in our database including the outcome of interest, as recommended previously [23].

### Statistical analysis

We applied a general linear regression model to estimate mean differences in common CIMT with 95% confidence intervals associated with risk factor clusters, adjusted for age, sex, diabetes mellitus, and history of CVD. Main results are additionally presented by sex and race-ethnicity. Potential differences in cluster relations between sexes, race-ethnic groups and age groups (<65y, 65-74y; <75) was evaluated using a multiplicative interaction term.

## Results

Our analysis included 59,025 individuals (47% women), originating from 14 cohorts. Overall, mean age was 58 years, 7% reported a previous history of cardiovascular disease, and 8,5% had diabetes. This group consisted of 46,377 Whites, 6876 Blacks, 2,206 Asians, and 2,575 Hispanics. The prevalence of elevated blood pressure was 33%, of elevated total cholesterol 67%, of overweight 64%, and of smoking 20% (Table 1). Mean common CIMT was 0.74 mm. Of the entire cohort, 14% had no risk factor, 38% one, 32% two, 14% three risk factors and 2% four risk factors.

**Fig 1:**
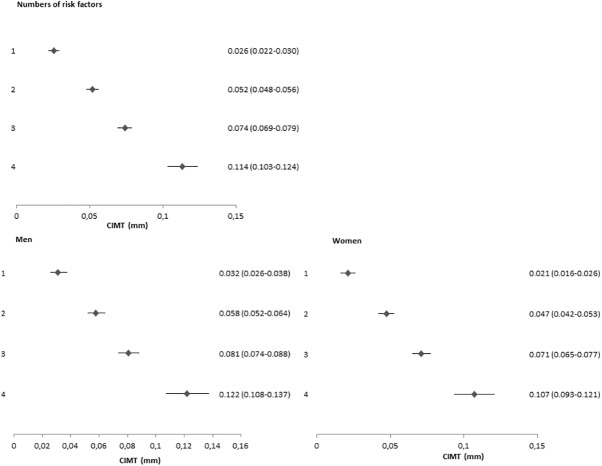
Relation between numbers of risk factors and difference in CIMT (Overall and by sex). Each number of risk factors was compared to individuals without any risk factor (reference group). CIMT, mean common carotid intima media thickness.

**Fig 2:**
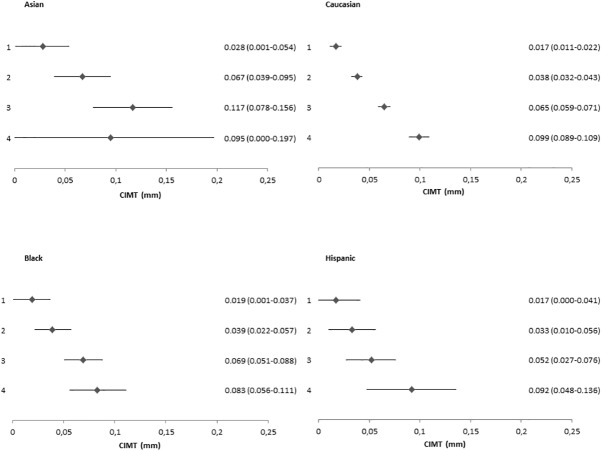
Relation between numbers of risk factors and differences in CIMT by race-ethnic group. **Each number of risk factors was compared to individuals without any risk factor (reference group). CIMT, mean common carotid intima media thickness.** Fig 3 presents the main findings of the overall analysis on risk factor clusters and CIMT. Within each risk factor cluster, there were graded relations with common CIMT. Within those with two risk factors, the cluster blood pressure-smoking had the highest CIMT (mean difference of 0.077 mm with those without risk factors) and the cluster with overweight- total cholesterol the least thickening (mean difference of 0.039 mm with those without risk factors), a difference reaching statistical significance with the cluster since the 95 confidence limits did not overlap. For people within the three risk factor cluster, elevated blood pressure, overweight and smoking had the highest common CIMT (0.084 mm). The pattern of the relationship between risk factor clusters and common CIMT were similar between sexes and race-ethnic groups, although some variation was observed between race-ethnic groups but was not significant due to limited minority samples sizes (S1 and S2 Figs). The interaction terms were not statistically significant.

The common CIMT increased with the number of risk factors present (Fig 1). Compared to those with no risk factors, those with one risk factor had a higher common CIMT (mean difference 0.026 mm (95%CI; 0.022, 0.030). For those with two, three and four risk factors, the increase in common CIMT was 0.052 mm (95%CI; 0.048,0.056), 0.074 mm (95%CI; 0.069,0.079) and 0.114 mm (95%CI; 0.103,0.124), respectively. Similar patterns were seen for men and women (Fig 1) and across race-ethnic groups (Fig 2).

**Fig 3:**
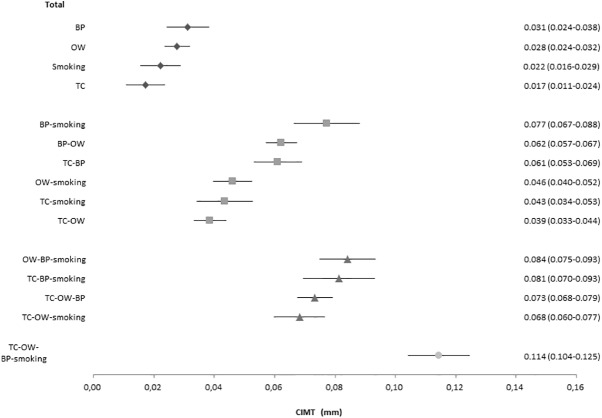
Relation between risk factor clusters and differences in CIMT (Overall). **Each cluster was compared to individuals without any risk factor (reference group).** CIMT, mean common carotid intima media thickness. BP, elevated blood pressure; OW, overweight; TC, elevated total cholesterol; smoking, current smoking.For most of the risk factors, the sum of the individual risk factor differences was smaller than the observed mean difference for the cluster in the overall analyses. For example, the mean difference in common CIMT for the blood pressure—smoking cluster was 0.077 mm, whereas the sum of the individual risk factors was 0.053 (i.e., 0.031 + 0.022). A similar finding was found for the smoking-blood pressure- overweight cluster. This observation suggests synergetic effects of risk factors on CIMT.

## Discussion

This study with 59,025 individuals found that clusters of modifiable risk factors act on common CIMT in a graded manner, in both men and women and across race-ethnic groups. The arterial wall thickness of people with multiple risk factors was generally higher than the sum of the parts, indicative of synergetic effects. Clusters including elevated blood pressure contributed most to the extent of atherosclerosis as measured with common CIMT.

Various national and international cardiovascular risk factor management guidelines promote assessment of absolute event risk based on a set of risk factors, and the absolute risk estimate drives the initiation of preventive therapy [28–30]. We expand that evidence base by showing the relevance of single and combined risk factor information with respect the extent of presence of atherosclerosis, i.e., the higher the CIMT value, the more extensive the atherosclerosis elsewhere in the arterial system [31,32]. Our study is in line with numerous population-based and hospital based studies which have shown graded relations between elevated levels of single modifiable risk factors and increased CIMT [33]. We have shown a graded relation when the burden of several risk factors increases, a finding similar for men as for women and similar across race-ethnic groups. Studies that addressed clustering of risk factors almost exclusively looked into the aspects of the metabolic syndrome, and showed increased CIMT within those with a metabolic syndrome. In contrast, we showed that some clusters have a more adverse effect on the development of atherosclerosis than others. Confirmation of the relation of several clusters with clinical events in this sense may be of great relevance to CVD prognosis.

The main impact of our findings is a further underpinning of the necessity of prevention of the development of key cardiovascular risk factors in order to address the ongoing and upcoming cardiovascular epidemic in high income societies and emerging economies, respectively. There is substantial evidence that addressing these modifiable risk factors through statin treatment [34,35], blood pressure treatment [36], smoking cessation[37] or interdisciplinary weight loss therapy[38] lead to halting the progression or even reduce common CIMT and thus slow down development of atherosclerosis. Such evidence complements the effects of risk factor management on reduction on cardiovascular events. Ways to improve cardiovascular health in communities in the future have been recently outlined by Vasan and co-worker pointing towards an approach that includes a full range of biological, environmental and social determinants of cardiovascular health[39]. Prevention strategies should indeed be targeted to the local situation, depending on the risk factor cluster prevalence (e.g. high in China, much lower in The Netherlands[40]), its associated risk with atherosclerosis and clinical events, cultural aspects regarding life style and prevention of the population and budgetary restraints.

The strength of our study comes from the large sample size from multiple cohorts from various parts of the world that collaborate in the ongoing USE-IMT initiative. Moreover, the cohorts in USE-IMT were derived from the general population, which increase the generalizability of our results. Furthermore, we analyzed the values of CIMT for sex and race-ethnic groups separately.

Several limitations should be emphasized when interpreting the results. First of all, due to the cross-sectional design we need to be cautious with causal interpretation. Yet, an increased CIMT at a certain age is viewed as the reflection of life long exposure to the cardiovascular risk factors. Also, it is well established that elevated blood pressure, smoking, overweight and elevated total cholesterol contributes to cardiovascular disease[1]. Furthermore, several reviews showed the beneficial effects of lipid lowering and blood pressure lowering on progression of common CIMT [41,42]. Secondly, one may argue that common CIMT in itself does not reflect atherosclerosis[43]. The measurement of common CIMT is, however, used as a reflection of atherosclerosis in the coronary arteries [44], the abdominal aorta [45], the arteries of the lower extremities, and in the carotid bifurcation and internal carotid artery [46]. In addition, common CIMT is a reflection of cardiovascular risk, as many studies have shown that increased common CIMT relates to an increased risk of cardiovascular events in the future [8]. Thirdly, the protocols to assess and measure mean common CIMT varied by the studies. This may have led to an increased variability of the common CIMT measurement and thus an underestimation of the relations under study.

In conclusion, clusters of modifiable risk factors are related to increased common CIMT, as an indicator of the extent of atherosclerosis, in a graded manner, similar in men, women and across race-ethnic groups. Some clusters seemed more atherogenic than others. Our findings support the notion that cardiovascular prevention should focus on sets of risk factors rather than individual levels alone, but may prioritize within clusters.

## Supporting information

S1 Fig Relation between risk factor clusters and CIMT (by sex). **Each cluster was compared to individuals without any risk factor (reference group).** CIMT, mean common carotid intima media thickness. BP, elevated blood pressure; OW, overweight; TC, elevated total cholesterol; smoking, current smoking.
(TIF)

S2 Fig Relation between risk factor clusters and CIMT (by race-ethnic group). **Each cluster was compared to individuals without any risk factor (reference group).** CIMT, mean common carotid intima media thickness. BP, elevated blood pressure; OW, overweight; TC, elevated total cholesterol; smoking, current smoking.
(TIF)

## References

[1] Forouzanfar MH, Alexander L, Anderson HR, Bachman VF, Biryukov S, Brauer M et al (2015) Global, regional, and national comparative risk assessment of 79 behavioural, environmental and occupational, and metabolic risks or clusters of risks in 188 countries, 1990-2013: a systematic analysis for the Global Burden of Disease Study 2013. Lancet 386: 2287–2323. 10.1016/S0140-6736(15)00128-2 26364544 PMC4685753

[2] O'Donnell MJ, Xavier D, Liu L, Zhang H, Chin SL, Rao-Melacini P et al (2010) Risk factors for ischaemic and intracerebral haemorrhagic stroke in 22 countries (the INTERSTROKE study): a case-control study. Lancet 376: 112–123. 10.1016/S0140-6736(10)60834-3 20561675

[3] Yusuf S, Hawken S, Ounpuu S, Dans T, Avezum A, Lanas F et al (2004) Effect of potentially modifiable risk factors associated with myocardial infarction in 52 countries (the INTERHEART study): case-control study. Lancet 364: 937–952. 10.1016/S0140-6736(04)17018-9 15364185

[4] Ross R, Glomset JA (1976) The pathogenesis of atherosclerosis (first of two parts). N Engl J Med 295: 369–377. 10.1056/NEJM197608122950707 819830

[5] McGill HC Jr., McMahan CA, Gidding SS (2008) Preventing heart disease in the 21st century: implications of the Pathobiological Determinants of Atherosclerosis in Youth (PDAY) study. Circulation 117: 1216–1227. 10.1161/CIRCULATIONAHA.107.717033 18316498

[6] Huxley RR, Barzi F, Woo J, Giles G, Lam TH, Rahimi K et al (2014) A comparison of risk factors for mortality from heart failure in Asian and non-Asian populations: an overview of individual participant data from 32 prospective cohorts from the Asia-Pacific Region. BMC Cardiovasc Disord 14: 61 10.1186/1471-2261-14-61 24884382 PMC4037783

[7] Rosvall M, Persson M, Ostling G, Nilsson PM, Melander O, Hedblad B et al (2015) Risk factors for the progression of carotid intima-media thickness over a 16-year follow-up period: the Malmo Diet and Cancer Study. Atherosclerosis 239: 615–621. 10.1016/j.atherosclerosis.2015.01.030 25746169

[8] den Ruijter HM, Peters SA, Anderson TJ, Britton AR, Dekker JM, Eijkemans MJ et al (2012) Common carotid intima-media thickness measurements in cardiovascular risk prediction: a meta-analysis. JAMA 308: 796–803. 10.1001/jama.2012.9630 22910757

[9] Li R, Duncan BB, Metcalf PA, Crouse JR III, Sharrett AR, Tyroler HA et al (1994) B-mode-detected carotid artery plaque in a general population. Atherosclerosis Risk in Communities (ARIC) Study Investigators. Stroke 25: 2377–2383. 7974576 10.1161/01.str.25.12.2377

[10] Lorenz MW, von KS, Steinmetz H, Markus HS, Sitzer M (2006) Carotid intima-media thickening indicates a higher vascular risk across a wide age range: prospective data from the Carotid Atherosclerosis Progression Study (CAPS). Stroke 37: 87–92. 10.1161/01.STR.0000196964.24024.ea 16339465

[11] Price JF, Tzoulaki I, Lee AJ, Fowkes FG (2007) Ankle brachial index and intima media thickness predict cardiovascular events similarly and increased prediction when combined. J Clin Epidemiol 60: 1067–1075. 10.1016/j.jclinepi.2007.01.011 17884603

[12] Anderson TJ, Charbonneau F, Title LM, Buithieu J, Rose MS, Conradson H et al (2011) Microvascular function predicts cardiovascular events in primary prevention: long-term results from the Firefighters and Their Endothelium (FATE) study. Circulation 123: 163–169. 10.1161/CIRCULATIONAHA.110.953653 21200002

[13] Henry RM, Kostense PJ, Spijkerman AM, Dekker JM, Nijpels G, Heine RJ et al (2003) Arterial stiffness increases with deteriorating glucose tolerance status: the Hoorn Study. Circulation 107: 2089–2095. 10.1161/01.CIR.0000065222.34933.FC 12695300

[14] Salonen R, Salonen JT (1991) Determinants of carotid intima-media thickness: a population-based ultrasonography study in eastern Finnish men. J Intern Med 229: 225–231. 2007840 10.1111/j.1365-2796.1991.tb00336.x

[15] Rosvall M, Ostergren PO, Hedblad B, Isacsson SO, Janzon L, Berglund G (2000) Occupational status, educational level, and the prevalence of carotid atherosclerosis in a general population sample of middle-aged Swedish men and women: results from the Malmo Diet and Cancer Study. Am J Epidemiol 152: 334–346. 10968378 10.1093/aje/152.4.334

[16] Mora S, Szklo M, Otvos JD, Greenland P, Psaty BM, Goff DC Jr et al (2007) LDL particle subclasses, LDL particle size, and carotid atherosclerosis in the Multi-Ethnic Study of Atherosclerosis (MESA). Atherosclerosis 192: 211–217. 10.1016/j.atherosclerosis.2006.05.007 16765964

[17] Holewijn S, den HM, Swinkels DW, Stalenhoef AF, de GJ (2009) The metabolic syndrome and its traits as risk factors for subclinical atherosclerosis. J Clin Endocrinol Metab 94: 2893–2899. 10.1210/jc.2009-0084 19417041

[18] Prabhakaran S, Singh R, Zhou X, Ramas R, Sacco RL, Rundek T (2007) Presence of calcified carotid plaque predicts vascular events: the Northern Manhattan Study. Atherosclerosis 195: e197–e201. 10.1016/j.atherosclerosis.2007.03.044 17482197 PMC6286814

[19] Halcox JP, Donald AE, Ellins E, Witte DR, Shipley MJ, Brunner EJ et al (2009) Endothelial function predicts progression of carotid intima-media thickness. Circulation 119: 1005–1012. 10.1161/CIRCULATIONAHA.108.765701 19204308

[20] Ali YS, Rembold KE, Weaver B, Wills MB, Tatar S, Ayers CR et al (2006) Prediction of major adverse cardiovascular events by age-normalized carotid intimal medial thickness. Atherosclerosis 187: 186–190. 10.1016/j.atherosclerosis.2005.09.003 16233899

[21] Stensland-Bugge E, Bonaa KH, Joakimsen O, Njolstad I (2000) Sex differences in the relationship of risk factors to subclinical carotid atherosclerosis measured 15 years later: the Tromso study. Stroke 31: 574–581. 10700488 10.1161/01.str.31.3.574

[22] Kitagawa K, Hougaku H, Yamagami H, Hashimoto H, Itoh T, Shimizu Y et al (2007) Carotid intima-media thickness and risk of cardiovascular events in high-risk patients. Results of the Osaka Follow-Up Study for Carotid Atherosclerosis 2 (OSACA2 Study). Cerebrovasc Dis 24: 35–42. 10.1159/000103114 17519542

[23] Gijsberts CM, Groenewegen KA, Hoefer IE, Eijkemans MJ, Asselbergs FW, Anderson TJ et al (2015) Race/Ethnic Differences in the Associations of the Framingham Risk Factors with Carotid IMT and Cardiovascular Events. PLoS One 10: e0132321 10.1371/journal.pone.0132321 26134404 PMC4489855

[24] den Ruijter HM, Peters SA, Groenewegen KA, Anderson TJ, Britton AR, Dekker JM et al (2013) Common carotid intima-media thickness does not add to Framingham risk score in individuals with diabetes mellitus: the USE-IMT initiative. Diabetologia 56: 1494–1502. 10.1007/s00125-013-2898-9 23568273 PMC4523149

[25] [Anonymous] (1995) Physical status: The Use and Interpretation of Anthropometry. WHO technical report serues 854. 8594834

[26] 1993) The fifth report of the Joint National Committee on Detection, Evaluation, and Treatment of High Blood Pressure (JNC V). Arch Intern Med 153: 154–183. 8422206

[27] 2002) Third Report of the National Cholesterol Education Program (NCEP) Expert Panel on Detection, Evaluation, and Treatment of High Blood Cholesterol in Adults (Adult Treatment Panel III) final report. Circulation 106: 3143–3421. 12485966

[28] Duerden M, O'Flynn N, Qureshi N (2015) Cardiovascular risk assessment and lipid modification: NICE guideline. Br J Gen Pract 65: 378–380. 10.3399/bjgp15X685933 26120133 PMC4484941

[29] Wiersma T, Smulders YM, Stehouwer CD, Konings KT, Lanphen J (2012) [Summary of the multidisciplinary guideline on cardiovascular risk management (revision 2011)]. Ned Tijdschr Geneeskd 156: A5104 22951134

[30] Piepoli MF, Hoes AW, Agewall S, Albus C, Brotons C, Catapano AL et al (2016) 2016 European Guidelines on cardiovascular disease prevention in clinical practice: The Sixth Joint Task Force of the European Society of Cardiology and Other Societies on Cardiovascular Disease Prevention in Clinical Practice (constituted by representatives of 10 societies and by invited experts)Developed with the special contribution of the European Association for Cardiovascular Prevention & Rehabilitation (EACPR). Eur Heart J 37: 2315–2381. 10.1093/eurheartj/ehw106 27222591 PMC4986030

[31] Bots ML, Baldassarre D, Simon A, de GE, O'Leary DH, Riley W et al (2007) Carotid intima-media thickness and coronary atherosclerosis: weak or strong relations? Eur Heart J 28: 398–406. 10.1093/eurheartj/ehl482 17277033

[32] Bots ML, Sutton-Tyrrell K (2012) Lessons from the past and promises for the future for carotid intima-media thickness. J Am Coll Cardiol 60: 1599–1604. 10.1016/j.jacc.2011.12.061 22999720

[33] Nair SB, Malik R, Khattar RS (2012) Carotid intima-media thickness: ultrasound measurement, prognostic value and role in clinical practice. Postgrad Med J 88: 694–699. 10.1136/postgradmedj-2011-130214 22761324

[34] Crouse JR III, Raichlen JS, Riley WA, Evans GW, Palmer MK, O'Leary DH et al (2007) Effect of rosuvastatin on progression of carotid intima-media thickness in low-risk individuals with subclinical atherosclerosis: the METEOR Trial. JAMA 297: 1344–1353. 10.1001/jama.297.12.1344 17384434

[35] Makris GC, Lavida A, Nicolaides AN, Geroulakos G (2010) The effect of statins on carotid plaque morphology: a LDL-associated action or one more pleiotropic effect of statins? Atherosclerosis 213: 8–20. 10.1016/j.atherosclerosis.2010.04.032 20494361

[36] Marfella R, Siniscalchi M, Nappo F, Gualdiero P, Esposito K, Sasso FC et al (2005) Regression of carotid atherosclerosis by control of morning blood pressure peak in newly diagnosed hypertensive patients. Am J Hypertens 18: 308–318. 10.1016/j.amjhyper.2004.09.013 15797646

[37] Pipe AL, Papadakis S, Reid RD (2010) The role of smoking cessation in the prevention of coronary artery disease. Curr Atheroscler Rep 12: 145–150. 10.1007/s11883-010-0105-8 20425251

[38] Masquio DC, de PA, Sanches PL, Corgosinho FC, Campos RM, Carnier J et al (2013) The effect of weight loss magnitude on pro-/anti-inflammatory adipokines and carotid intima-media thickness in obese adolescents engaged in interdisciplinary weight loss therapy. Clin Endocrinol (Oxf) 79: 55–64. 22809141 10.1111/j.1365-2265.2012.04504.x

[39] Vasan RS, Benjamin EJ (2016) The Future of Cardiovascular Epidemiology. Circulation 133: 2626–2633. 10.1161/CIRCULATIONAHA.116.023528 27324358 PMC4974092

[40] Wang X, Bots ML, Yang F, Sun J, He S, Hoes AW et al (2016) A comparison of the prevalence and clustering of major cardiovascular risk factors in the Netherlands and China. Eur J Prev Cardiol. 10.1177/2047487316648474 27154590

[41] Wang JG, Staessen JA, Li Y, Van Bortel LM, Nawrot T, Fagard R et al (2006) Carotid intima-media thickness and antihypertensive treatment: a meta-analysis of randomized controlled trials. Stroke 37: 1933–1940. 10.1161/01.STR.0000227223.90239.13 16763185

[42] Peters SA, Lind L, Palmer MK, Grobbee DE, Crouse JR III, O'Leary DH et al (2012) Increased age, high body mass index and low HDL-C levels are related to an echolucent carotid intima-media: the METEOR study. J Intern Med 272: 257–266. 10.1111/j.1365-2796.2011.02505.x 22172243

[43] Bots ML, Hofman A, Grobbee DE (1997) Increased common carotid intima-media thickness. Adaptive response or a reflection of atherosclerosis? Findings from the Rotterdam Study. Stroke 28: 2442–2447. 9412629 10.1161/01.str.28.12.2442

[44] Bots ML, Baldassarre D, Simon A, de GE, O'Leary DH, Riley W et al (2007) Carotid intima-media thickness and coronary atherosclerosis: weak or strong relations? Eur Heart J 28: 398–406. 10.1093/eurheartj/ehl482 17277033

[45] Bots ML, Hofman A, de Bruyn AM, De Jong PT, Grobbee DE (1993) Isolated systolic hypertension and vessel wall thickness of the carotid artery. The Rotterdam Elderly Study. Arterioscler Thromb 13: 64–69. 8422340 10.1161/01.atv.13.1.64

[46] Bots ML, Hofman A, De Jong PT, Grobbee DE (1996) Common carotid intima-media thickness as an indicator of atherosclerosis at other sites of the carotid artery. The Rotterdam Study. Ann Epidemiol 6: 147–153. 8775595 10.1016/1047-2797(96)00001-4

