# Supplementary figures and images for: Clustering of cardiovascular risk factors and carotid intima-media thickness: The USE-IMT study
Source: PLoS One. 2017 Mar 21;12(3):e0173393. doi: 10.1371/journal.pone.0173393 (PMC5360240; doi:10.1371/journal.pone.0173393)

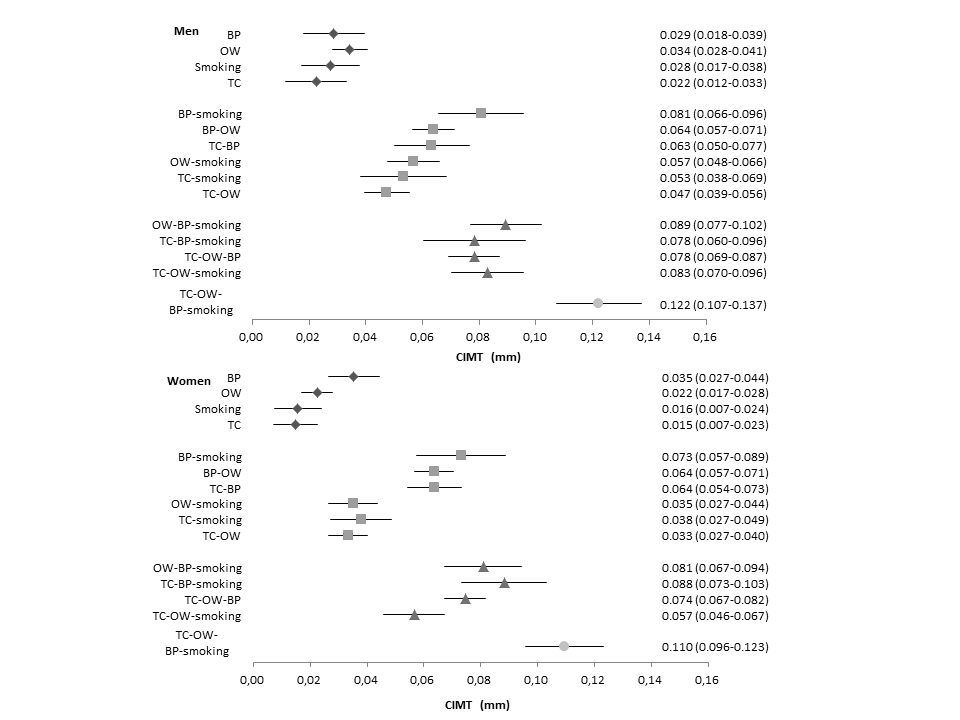

Supplement: S1 Fig — Each cluster was compared to individuals without any risk factor (reference group). CIMT, mean common carotid intima media thickness. BP, elevated blood pressure; OW, overweight; TC, elevated total cholesterol; smoking, current smoking. (TIF) [file pone.0173393.s001.tif]

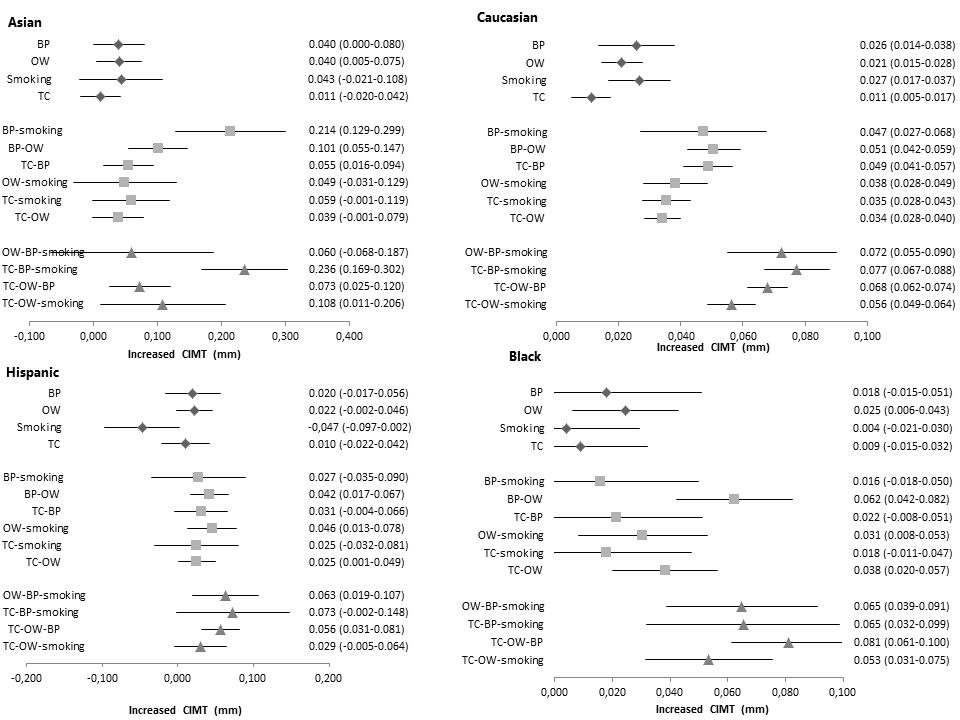

Supplement: S2 Fig — Each cluster was compared to individuals without any risk factor (reference group). CIMT, mean common carotid intima media thickness. BP, elevated blood pressure; OW, overweight; TC, elevated total cholesterol; smoking, current smoking. (TIF) [file pone.0173393.s002.tif]
